# *Stevia rebaudiana* Bertoni responses to salt stress and chitosan elicitor

**DOI:** 10.1007/s12298-020-00788-0

**Published:** 2020-04-16

**Authors:** Mahyar Gerami, Parastoo Majidian, Akram Ghorbanpour, Zeinab Alipour

**Affiliations:** 1 Department of Biology, Sana Institute of Higher Education, Sari, Iran; 2 Crop and Horticultural Science Research Department, Mazandaran Agricultural and Natural Resources Research and Education Center, Agricultural Research, Education and Extension Organization (AREEO), Sari, Iran; 3 Department of Plant Biology, Kharazmi University, Tehran, Iran

**Keywords:** *Stevia rebaudiana*, Chitosan elicitor, Salt stress, HPLC

## Abstract

This study examined the effect of chitosan elicitor with four different concentrations (0, 0.2, 0.4 and 0.6 g/l) on physiological and biochemical properties of stevia under four levels of salinity stress (0, 50, 100, 150 mM level of NaCl). Salt stress caused reduction of chlorophyll *a* (Chl *a*), chlorophyll *b* (Chl *b*), total chlorophyll, carotenoid and total protein content. The increment of malondialdehyde (MDA) content was not significant in all NaCl levels, while the CAT and POX activities were increased as well as stevioside and rebaudioside A under salinity stress. On one side, chitosan treatments could compensate the reduction of physiological traits such as photosynthetic pigments and protein content. On the other side, chitosan caused multiple increases in malondialdehyde content, antioxidant enzymes activity (catalase and peroxidase), steviol glycosides (stevioside and rebaudioside A) under salt stress. We report for the first time, the potential of chitosan to enhance salinity-tolerant abilities in stevia through increment of the salt-adaptive factors and to diminish harmful damages caused by NaCl stress.

## Introduction

Stevia (*Stevia rebaudiana* Bertoni) is an herbaceous and perennial medicinal plant, belongs to the Asteraceae family and native to Paraguay and Brazil (Pal et al. 2013; Halim et al. 2016). Stevia is commonly used as natural sweetener to control diabetes and other related diseases due to the presence of diterpenoid steviol glycosides (stevioside and rebaudioside A). This type of carbohydrate is considered to be about 300 times sweeter than sucrose which causes stevia to be known as sugar leaf or sweet leaf. The body does not metabolize the glycosides in stevia, so it contains zero calories like some artificial sweeteners (Kumar et al. 2013).

Soil salinity conditions have known to be a major constraint in more than one hundred hectares of agricultural lands in Iran (Emadodin et al. 2012). The significant destructive effects of salt stress are on growth, development, differentiation levels as well as primary metabolisms including photosynthesis, protein synthesis, energy production and lipid metabolism in plants (Acosta-Motos et al. 2015; Wu 2018). To solve this matter, enhancement of capability of plants in order to preserve their growth and yield under salt stress condition, followed by introduction of salt-tolerant cultivars are of great importance (Munns et al. 2002).

Recently, researchers have focused on utilization of elicitors with biological or non-biological nature to induce chemical defense system of plants aiming at high yield productivity of secondary metabolites under abiotic and biotic stresses (Samota et al. 2017; Mejía-Espejel et al. 2018). Chitosan (CHT), a polysaccharide (C_6_H_11_O_4_N), is one of biological elicitor obtained from naturally de-acetylation of chitin which is present in diverse organisms such as fungi, mollusks, diatoms, marine and fresh water sponges (Kurita 2006). Its antimicrobial characteristics against numerous bacteria, viruses and fungi make it useable as a thin layer on fruits and vegetables to prevent premature decay (Romanazzi et al. 2018).

Chitosan has numerous advantageous such as being safe, inexpensive and easily correlated to other compounds for better achievement (Malerba and Cerana 2016; Bistgani et al. 2017). Application of chitosan in agriculture was shown to enhance physiological responses such as induction of defense enzymes and synthesis of secondary metabolites consisting of polyphenolics, lignin, flavonoids, and phytoalexins in many plant species such as papaya (Ali et al. 2011), tomato (Badawy and Rabea 2009; Sharma et al. 2010), sunflower (Cho et al. 2008), apricot (Ghasemnezhad and Shiri 2010), soybean (Khan et al. 2002), *Chenopodium quinoa* (Mansouri and Omidi 2018), rice (Martínez et al. 2015), grape (Meng et al. 2008), spinach (Singh 2016) and wheat (Zeng and Luo 2012). Besides numerous physiological activities of chitosan, it has also been reported that it can improve growth and yield of plant (Cho et al. 2008).

In case of interaction of salinity and chitosan elicitor, few studies have been reported on association of chitosan and salinity stress on various properties of plants such as tomato (Hernández-Hernández et al. 2018) and chickpea (Mahdavi and Safari 2015). However, no literature is available on usage of chitosan elicitor for improvement of morphological and physiological properties of stevia. Therefore, the aim of this study was to investigate the effect of this elicitor on several parameters of stevia including chlorophyll contents, carotenoid content, total protein content, antioxidant metabolisms (CAT and POX), oxidative stress parameter (lipid peroxidation/MDA content) and steviol glycoside contents (stevioside and rebaudioside A) under salinity stress.

## Materials and methods

### Experimental design and plant material

This study was carried out based on completely randomized design with two treatments, different levels of salinity (0, 50, 100 and 150 mM NaCl) and different concentrations of chitosan elicitor (0, 0.2, 0.4 and 0.6 g/l) with three replications at greenhouse of Sana Institute of Higher Education of Sari-Iran (2016–2017). The seeds of *Stevia rebaudiana* L. were sown in trays containing sand, potting mix and cocopeat; 4:3:3 v/v, respectively. Fifteen days after germination, the three seedlings of stevia were transplanted into the pots (30 × 30 cm) containing the mixture of perlite and peat moss (ratio of 50:50). The growth condition was prepared as 16 h of light and 8 h of darkness at temperature of 25 °C with relative humidity of 70–80%. To nourish each sample, each pot was watered twice a week with 500 ml per pot of modified Hoagland solution. To apply the salinity treatments, the NaCl solutions were added 10 days after transplanting until the end of the experiment. In addition, chitosan elicitor was sprayed on each seedling twice the same days as salinity treatments. After the last treatment, the whole plants were harvested at 60 days after transplanting and leaves were separated and were placed in the freezer to measure the physiological and biochemical factors.

### Chlorophylls and carotenoid

The chlorophylls (chlorophyll *a*, *b* and total chlorophyll) and carotenoid contents were measured based on the method of Lichtenthaler and Wellburn (1983). This method was performed by extracting the fresh tissue (0.5 g) using 4 ml of 80% acetone. To estimate Chl *a*, Chl *b* and carotenoid contents, the optical density (O.D.) of each extract was examined using UV/VIS spectrophotometer at wavelengths of 663.2, 646.8 and 470 nm, respectively. The following equations were used to measure the amount of pigment.

$${\text{Chl}}\,a\,\left( {{\text{mg}}/{\text{g}}\,{\text{FW}}} \right) = 12.25 \times {\text{A}}_{663.2} - 2.79 \times {\text{A}}_{646.8}$$

$${\text{Chl}}\,b\,\left( {{\text{mg/g}}\,{\text{FW}}} \right) = 21.51 \times {\text{A}}_{646.8} - 5.1 \times {\text{A}}_{663.2}$$

$${\text{Chl}}\,\left( {a + b} \right) = {\text{Chl}}\,a + {\text{Chl}}\,b$$

$${\text{Carotenoid}}\,\left( {{\text{mg/g}}\,{\text{FW}}} \right) = \left( {1000 \times {\text{A}}_{470} {-}1.8 \times {\text{Chl}}\,a{-}85.52 \times {\text{Chl}}\,b} \right)/198$$

### Glycosides quantification

The quantification of stevioside and rebaudioside A were performed by HPLC (unicam-crystal-200, England) with a diode array detector based on the method of Martins et al. (2016). The main part of mobile phase consisted of acetonitrile (80%) buffered to pH = 0.5 with 100 ml of 0.02 M glacial acetic acid and 200 µl of 0.1 M sodium hydroxide for every 500 ml of the total solvent. The injection volume of 5 μl and a constant flow rate of 0.7 ml/min were programmed to flow through the Agilent Zorbax column (250 × 4.6 mm, 5 μm) in gradient mode varying from 10:90 to 90:10 v/v following the detection by UV at 210 nm.

### Total protein content

The concentration of protein in stevia extracts was evaluated using the Bradford method (1976). The dried leaves of samples were homogenized by 2 ml of phosphate potassium buffer (pH = 6.8). The samples were centrifuged at 15,000 rpm for 15 min following determination of protein concentration based on the BSA standard curve which is displayed as mg of protein extracted from 1 g of dried stevia leaves.

### Catalase

The procedure of catalase enzyme activity was carried out based on Chance and Maehly (1955). In this case, 20 µl of enzyme extract was mixed with 980 µl of phosphate buffer including 2 mM H_2_O_2_, and then the absorbance of the mixture was read at 240 nm by UV/VIS spectrophotometer.

### Peroxidase

For peroxidase activity, the reaction mixture contained 2.90 ml of 0.03% H_2_O_2_ (substrate) in 0.01 M phosphate buffer (pH = 6.0), 25 µl of 1% ortho-dianisidine in methanol and 100 µl of enzyme extract. The reaction was initiated by addition of the respective enzyme. The change in colour of oxidized dye was read at 460 nm up to 1 min at an interval of 15 s. Blank was prepared without addition of substrate. The enzyme activity was expressed as change in O.D./min/g of fresh tissue (Jabeen and Ahmad 2013).

### Lipid peroxidation of membrane assay

The lipid peroxidation experiment was measured on the basis of malondialdehyde (MDA) concentration and other aldehydes (Valentovic et al. 2006). Based on this method, 0.2 g of fresh leaf’s tissue was weighed and ground in 5 ml of 0.1% trichloroacetic acid (TCA), followed by centrifugation of 10,000*g* for 5 min. Then, 4 ml of 20% trichloroacetic acid (TCA) consisting of 0.5% thiobarbituric acid (TBA) was added to 1 ml of supernatant after centrifugation. The absorbance of this solution was evaluated by UV/VIS spectrophotometer at 532 nm.

### Statistical analysis

All experiments were set up in a factorial arrangement using completely randomized design with three replications. Analysis of variance was carried out by using SAS software version 9.4 and mean comparison of Duncan test was performed at 1% and 5% of probability level.

## Results

### Photosynthetic pigments

**Fig. 1:**
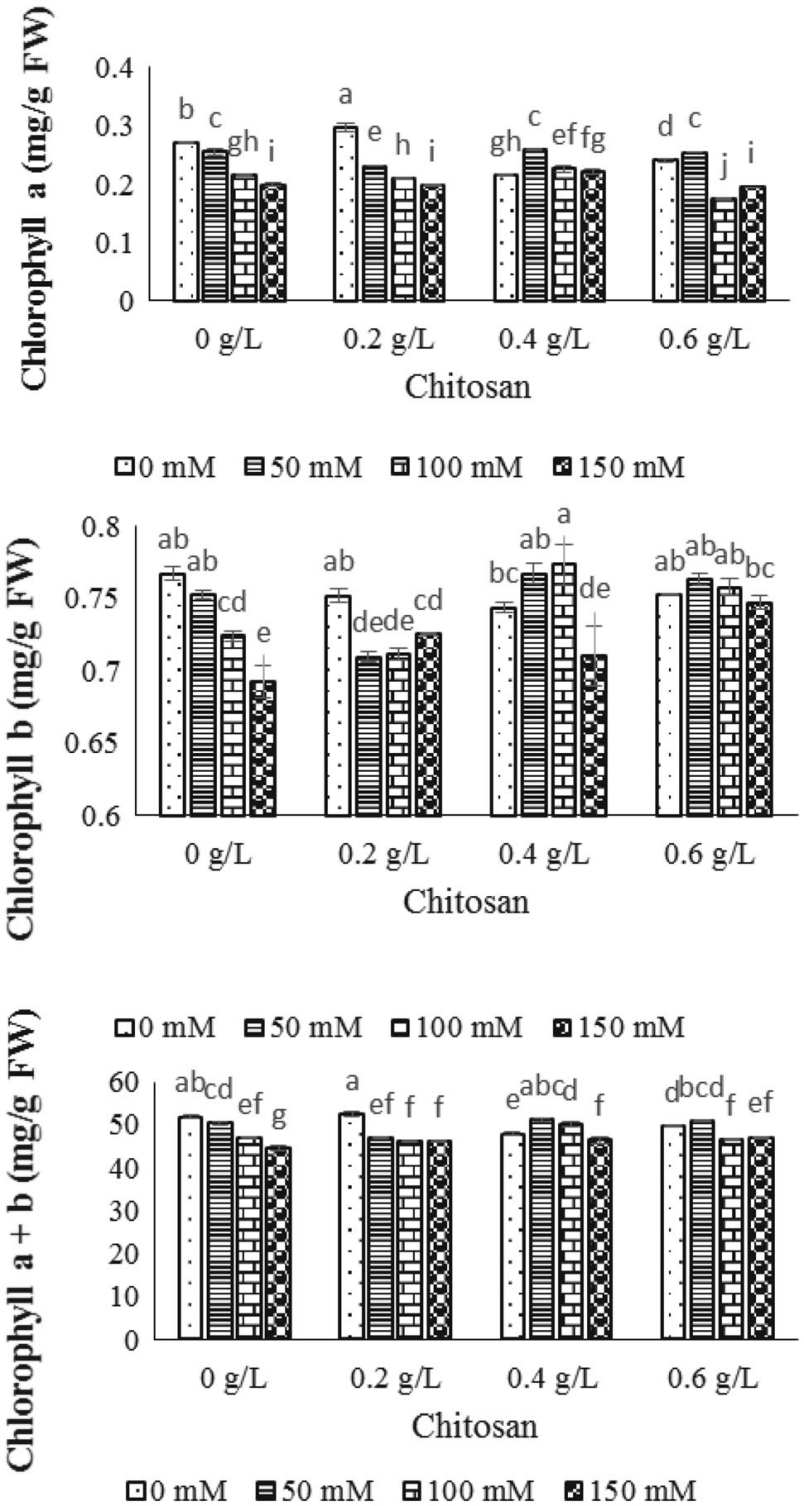
The effect of NaCl × chitosan interaction on chlorophyll *a*, chlorophyll *b* and total chlorophyll of leaf extract of *Stevia rebaudiana*

Based on ANOVA analysis, the chitosan × salt interaction effect on traits including (chlorophyll *a*, chlorophyll *b*, total chlorophyll and carotenoid) was significant at probability level of 1% (Table 1). The photosynthetic pigments (Chl *a*, Chl *b* and total Chl) decreased by NaCl treatments (Fig. 1). In 50 mM level of NaCl, the contents of chlorophyll *a*, chlorophyll *b* and total chlorophyll were increased by using 0.4 g/l and 0.6 g/l chitosan (Fig. 1). Thus, the interaction of low level of NaCl (50 mM) and high concentration of chitosan (0.4 and 0.6 g/l) resulted in greater increase rate in the traits studied compared to the control sample. Application of 0.4 g/l chitosan could exhibit the highest amount of all trait studied in 100 mM level of NaCl. In 150 mM of sodium chloride, all of elicitor concentrations had no effect on chlorophyll *b* and total chlorophyll, whereas chlorophyll *a* was increased by 0.4 g/l chitosan in 150 mM NaCl. In addition, no increment was shown in all photosynthetic contents studied by interaction effect of 0.2 g/l chitosan in all levels of NaCl (Fig. 1).

**Table 1:** Mean comparison of the traits studied in stevia’s leaf

SOV	*df*	Chl *a*	Chl *b*	Total chl	Carotenoid	Stevioside	Rebaudioside A	Total protein	Catalase	Peroxidase	Lipid peroxidation
Chitosan	3	0.00080030**	0.00253267**	2.0666920*	0.12952026**	123.5744549**	146.8165891**	164.391273**	957839.99**	41313.7269**	1.11075593**
NaCl	3	0.00963527**	0.00255254**	50.9571989**	1.36905543**	78.3183679**	26.6454757**	332.032635**	756600.25**	8989.3337**	0.77297788**
Chitosan × NaCl	9	0.00167560**	0.00126672**	11.3683150**	0.60718680**	47.5795366**	18.4688959**	777.480113**	1751380.47**	50533.5566**	1.01785902**
Error	32	0.00001936	0.00003074	0.0088517	0.00050562	0.065330	0.0050138	0.000454	0.01	0.0052	0.00639060
CV		1.919139	0.750429	0.194193	0.447850	0.916061	0.436870	0.087093	0.008634	0.015451	5.155973

* and ** shows significant effect at probability level of 5% and 1%

**Fig. 2:**
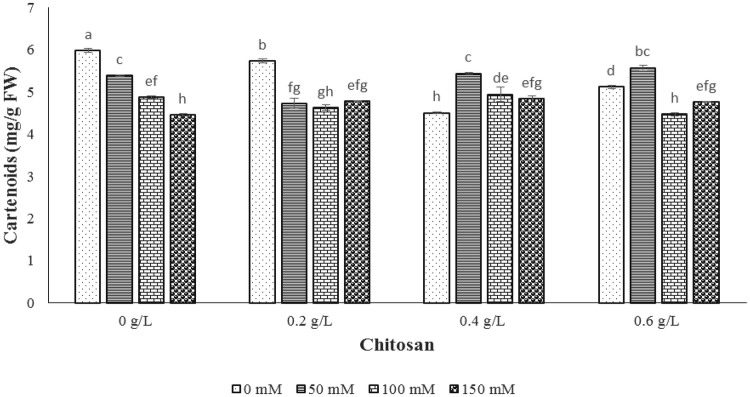
The effect of NaCl × chitosan interaction on carotenoid content of leaf extract of *Stevia rebaudiana*

Concerning the carotenoid result, the NaCl levels reduced carotenoid content compared to the control sample. In 50 mM of NaCl level, 0.2 g/l and 0.6 g/l of chitosan caused the reduction and enhancement rate of 12.2% and 3.5% in carotenoid content, respectively (Fig. 2). In level of 100 mM sodium chloride, no significant change was indicated by 0.4 g/l of chitosan, however, 0.2 and 0.6 g/l of chitosan resulted in increase of carotenoid content as 5.1% and 8.3%, respectively. Moreover, the highest carotenoid content was gained by 0.4 g/l of chitosan treatment in 150 mM level of sodium chloride compared to control sample (Fig. 2).

### Diterpene glycosides

**Fig. 3:**
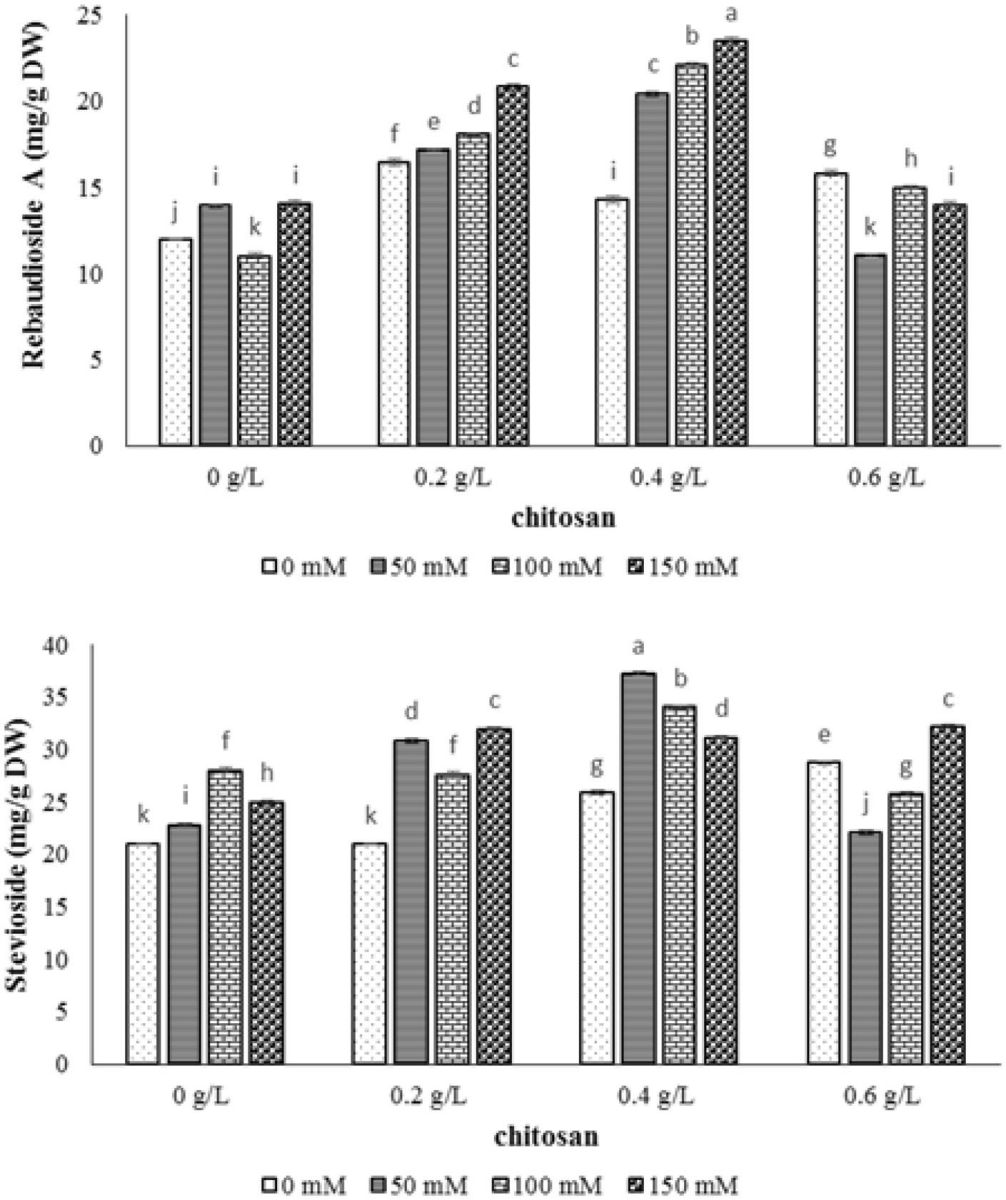
The effect of NaCl × chitosan interaction on stevioside and rebaudioside A of leaf extract of *Stevia rebaudiana*

The interaction effect of chitosan and salt stress was significant on steviol glycosides (stevioside and rebaudioside A) contents at probability level of 1% (Table 1). The stevioside content increased by NaCl treatment up to 100 mM and then showed a decrease (Fig. 3). However, in 50 and 100 mM of NaCl level, the application of 0.4 g/l chitosan indicated the highest level of stevioside content. In 150 mM level of NaCl, the utilization of 0.2 and 0.6 g/l of chitosan showed the greatest content of stevioside (Fig. 3). In the case of rebaudioside A, the use of 0.4 g/l of chitosan caused the highest amount of this glycoside in all levels of NaCl (50, 100 and 150 mM of NaCl). Our data showed that the chitosan concentration of 0.6 g/l showed no increase in the amount of rebaudioside A compared to the control sample. In addition, the results showed that the trend of stevioside and rebaudioside A contents were almost opposite at 4 g/l chitosan in all NaCl treatments (Fig. 3). Shibata et al. (1991) reported that rebaudioside A can be produced from stevioside substrate which probably leads to higher level of rebaudioside A and low stevioside in *S. rebaudiana* plants. The results indicated that chitosan could modulate the composition of steviol glycosides for its function of promoting the transformation of stevioside to rebaudioside A.

### Total protein content

**Fig. 4:**
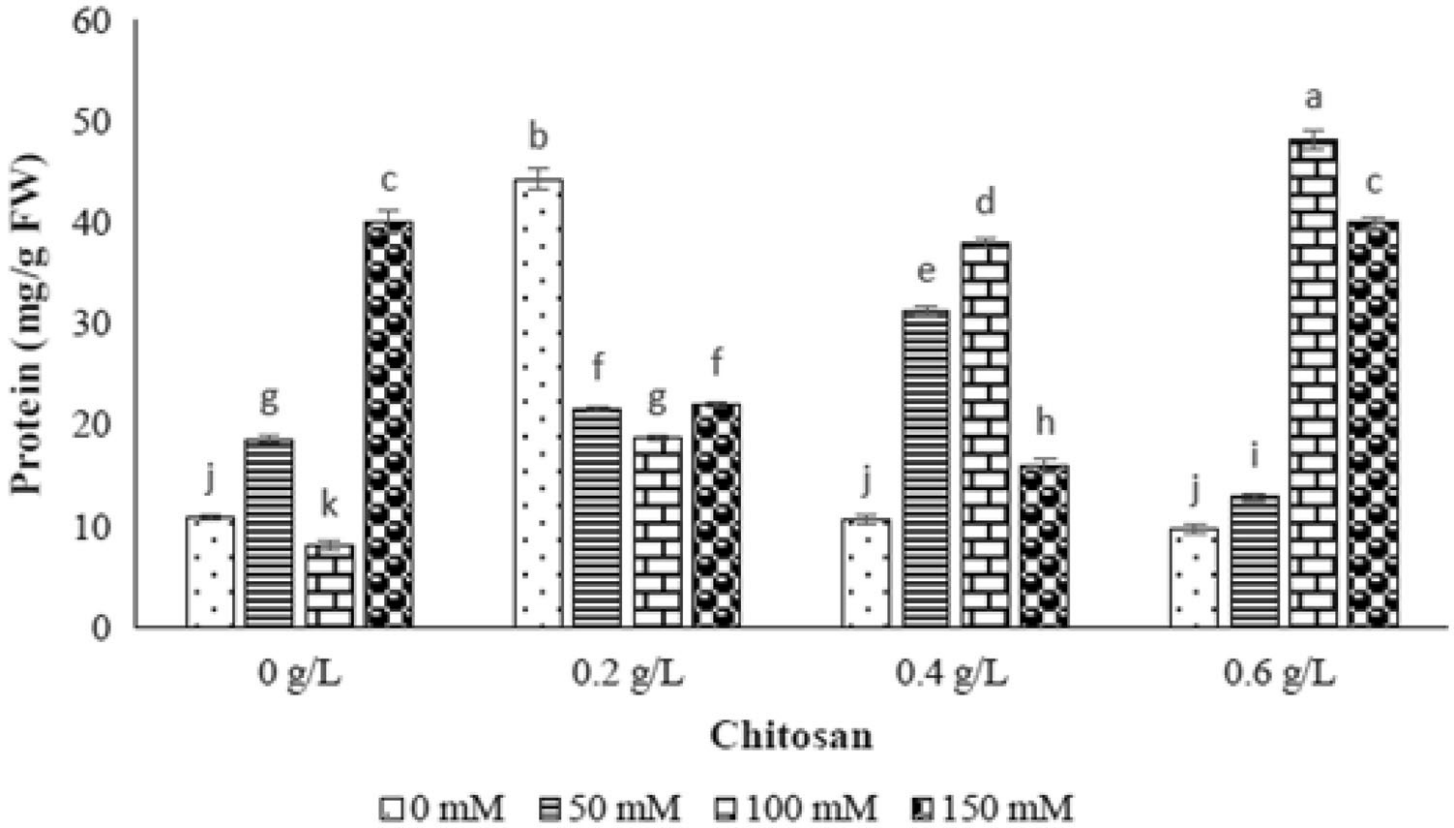
The effect of NaCl × chitosan interaction on total protein of leaf extract of *Stevia rebaudiana*

The NaCl effect was significant on the total protein content of stevia at probability level of 1% (Table 1). As shown in Fig. 4, the increase of NaCl concentration caused significantly enhancement protein content in terms that the 71.6% and 269.1% increase of protein content were exhibited in 50 and 150 mM levels of sodium chloride, respectively and the reduction of 26.3% was considered in 100 mM level of NaCl compared to the lowest level of salinity control (Fig. 4). According to addition of chitosan elicitor, the concentration of 0.2 g/l of chitosan showed no increase on protein content in different levels of salinity compared to the control sample. However, the chitosan elicitor treatment (0.6 g/l) caused the highest rise in protein content in 100 and 150 mM of NaCl, and the utilization of 0.4 g/l of chitosan caused the highest content of total protein in 50 mM level of NaCl (Fig. 4).

### Catalase enzyme (CAT)

**Fig. 5:**
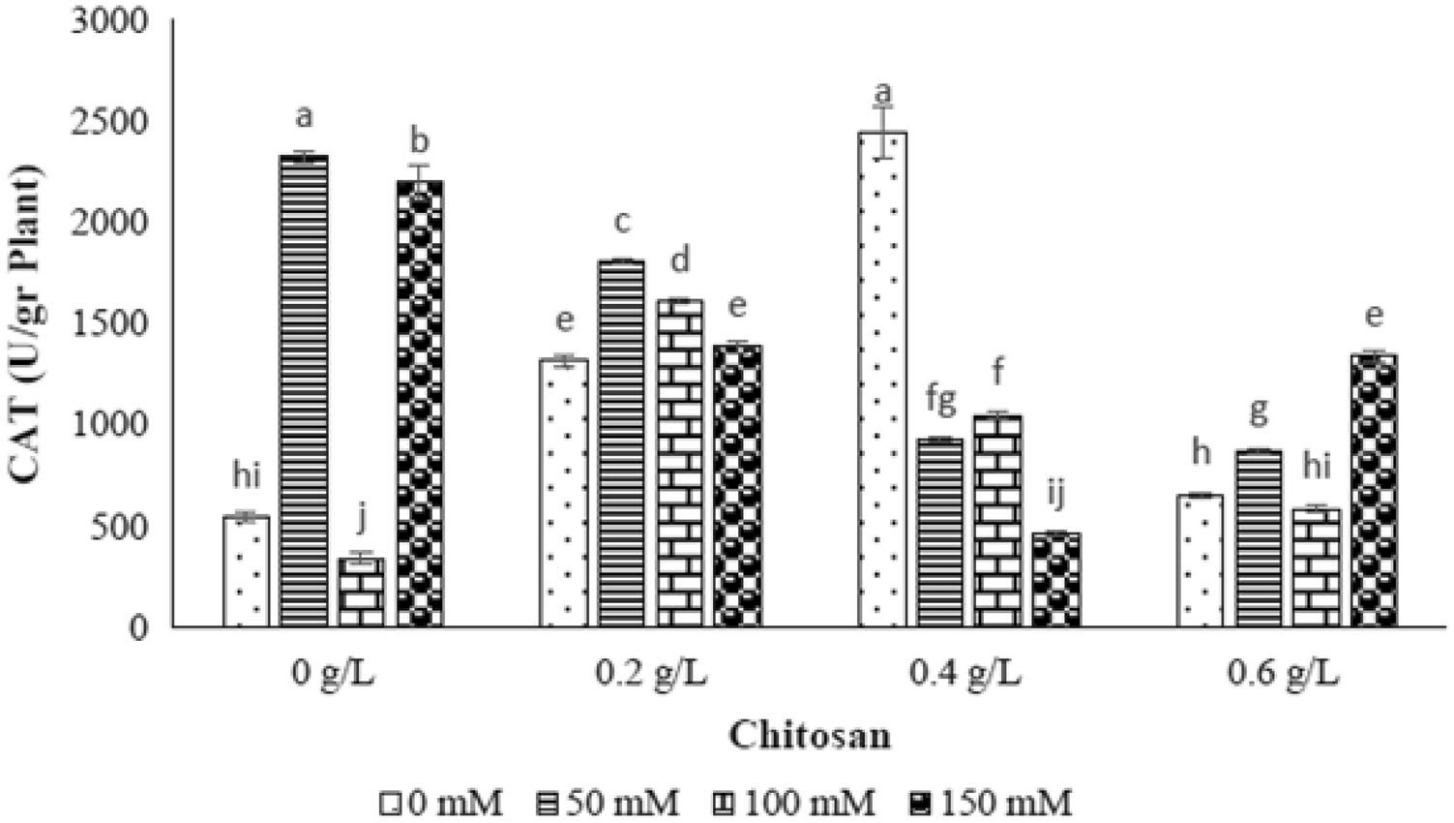
The effect of NaCl × chitosan interaction on CAT activity of leaf extract of *Stevia rebaudiana*

The significant different of catalase enzyme activity was gained by different levels of NaCl (*p* ≤ 0.01) (Table 1). The catalase activity was raised by increasing the NaCl level as 50 mM (327%) and 150 mM (303.3%), however, the reduction rate of enzyme activity was 37.3% in 100 mM of NaCl (Fig. 5). With respect to interaction effect of chitosan and elicitor results, the highest catalase enzyme activity was indicated by 0.2 g/l of chitosan-50 mM level of NaCl, 0.2 g/l of chitosan-100 mM level of NaCl and 0.6 g/l of chitosan-150 mM level of NaCl. While, the 0.4 g/l of chitosan was not able to increase the catalase enzyme activity in all levels of NaCl (Fig. 5).

### Peroxidase enzyme (POX)

**Fig. 6:**
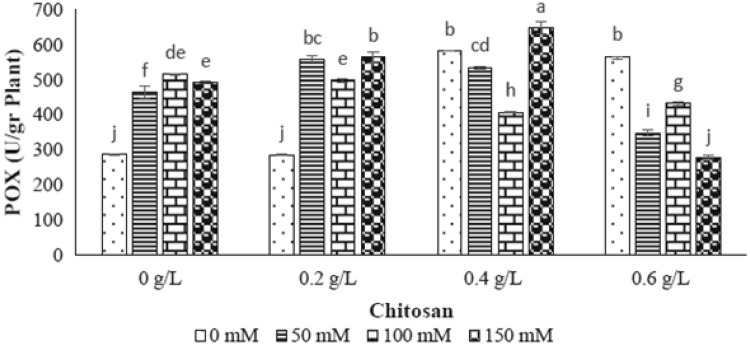
The effect of NaCl × chitosan interaction on POX activity of leaf extract of *Stevia rebaudiana*

Different levels of NaCl had significant effect on peroxidase enzyme activity as shown in Table 1. The increasing level of NaCl (50, 100 and 150 mM) resulted in enhancement of this enzyme (61%, 79% and 71%), respectively (Fig. 6). In 50 and 100 mM of NaCl level, the only 0.2 g/l of chitosan showed the greatest increase of this enzyme activity compared to control sample, while the treatment of 0.4 and 0.6 g/l chitosan resulted in reduction of 21% and 16% in POX activity, respectively. In 150 mM level of NaCl, the elevated peroxidase enzyme activity was achieved by application of 0.4 g/l of chitosan (Fig. 6).

### Lipid peroxidation

**Fig. 7:**
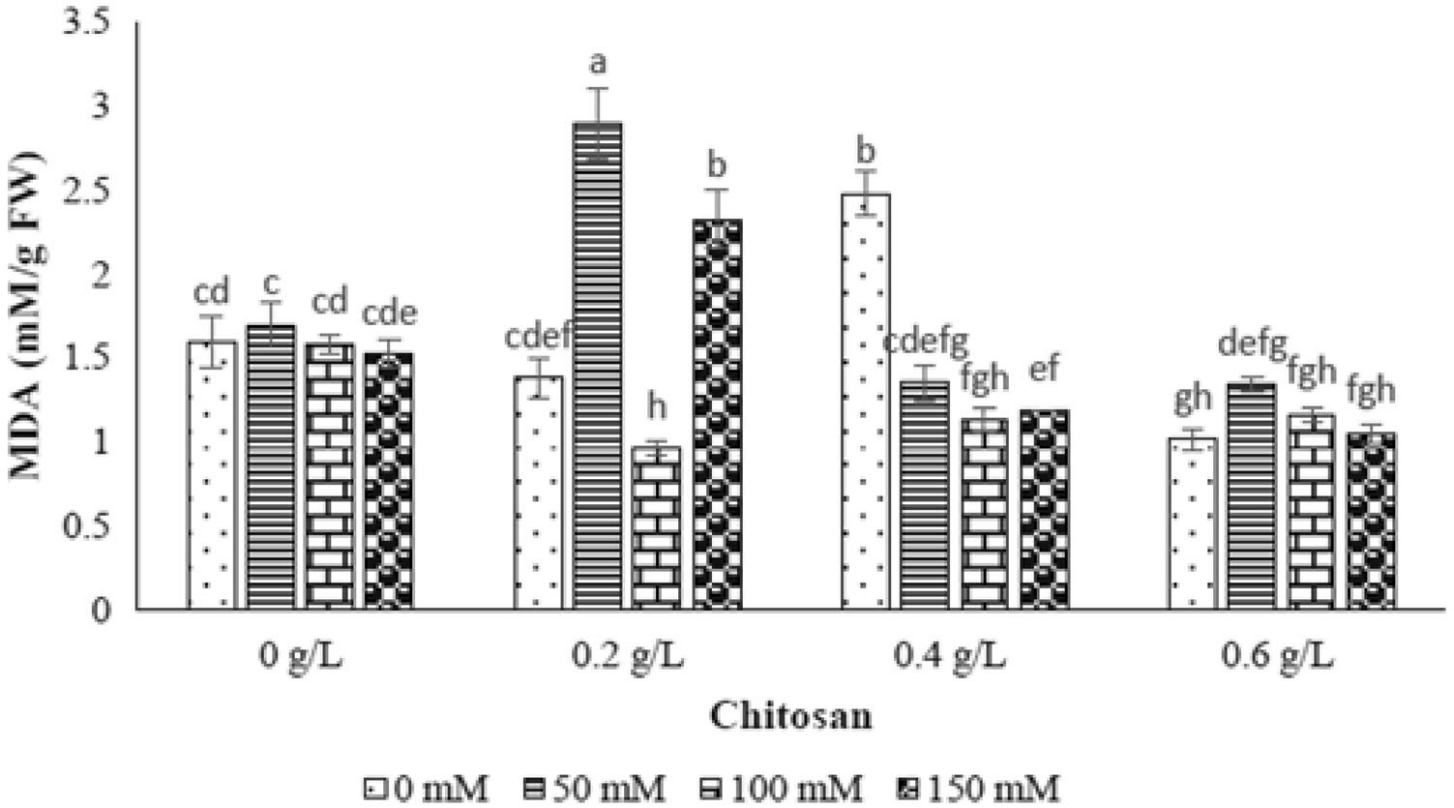
The effect of NaCl × chitosan interaction on lipid peroxidation of leaf extract of *Stevia rebaudiana*

Based on ANOVA analysis, the salt stress levels was not significant on lipid peroxidation (Table 1). As shown in Fig. 7, no significant increase of lipid peroxidation was observed in all level of NaCl (Fig. 7). However, the interaction effect of chitosan and salt stress showed significant difference on MDA content (Fig. 7). In this case, in 50 mM level of NaCl, the MDA content was incremented by using 0.2 g/l and 0.6 g/l of chitosan, respectively. The application of 0.4 g/l of chitosan couldn’t effect enhancement of MDA content in all levels of NaCl. In 150 mM level of NaCl, the only treatment of 0.2 g/l of chitosan indicated increase of MDA content, while the other chitosan treatments reduced the trait studied (Fig. 7). As a result, the chitosan concentrations of 0.2, 0.6 and 0.2 g/l showed the highest level of MDA content in 50, 100 and 150 mM level of NaCl, respectively (Fig. 7).

## Discussion

In the present study, the chlorophyll *a*, *b*, total chlorophyll and carotenoid contents were decreased by NaCl stress which was compatible with the results of previous studies (Rathore et al. 2014; Zeng et al. 2013) reported on negative effect of NaCl on photosynthetic pigments content. It is assumed that some factors including the lack of potassium and magnesium ions, the decline of potassium to sodium proportion, the destruction of chlorophyll structure by increase the chlorophyllase activity and the ultrastructure change of organelles and pigments could cause decline in chlorophyll content (Ashraf and Harris 2013).

In order to make up the acquired decline in photosynthetic pigments contents, different concentrations of chitosan elicitor (0, 0.2, 0.4 and 0.6 g/l) were used in four levels of NaCl (0, 50, 100 and 150 mM). The resultant data showed that 0.4 and 0.6 g/l of chitosan treatments in 50 mM level of NaCl could be the best choice for increasing chlorophylls and carotenoid contents, following by photosynthesis process. In previous study, the effect of foliar application of chitosan with different concentrations was assessed on growth and chlorophyll content of edible rape (*Brassica rapa* L.) in hydroponic pot experiments, resulting in reduction of toxic effects of cadmium, followed by increase of plant growth and lead to increase of chlorophyll content (Zong et al. 2017). Furthermore, in another study, the increment of chlorophyll and carotenoids contents was detected in *Phaseolus vulgaris* by addition of chitosan nanoparticle (Espirito Santo Pereira et al. 2017).

Steviol glycoside is a group of secondary metabolites derived from the mono-, di- and tetra-terpene biosynthetic pathway. The major steviol glycosides as stevioside and rebaudioside A are non-caloric sweeteners that are used in many countries due to being sweeter than sucrose (Ceunen and Geuns 2013). According to the results of diterpenoid steviol glycosides, the accumulation of glycosides was observed by increasing the levels of NaCl (Fig. 3). The same result was obtained by Shahverdi et al. (2019) which recorded the increase of steviol glycosides content under NaCl stress in stevia. In other study, the stevioside and rebaudioside A were not affected by low levels of salt (Cantabella et al. 2017). In addition, Zeng et al. (2013) reported that 90–120 mM NaCl treatments decreased the content of rebaudioside A and stevioside by 16.2–38.2%, while rebaudioside A and stevioside were promoted and reduced significantly in 60 mM NaCl. It has been reported that these metabolites function as osmoprotectant molecules under stress conditions (Cantabella et al. 2017). To raise the amount of these molecules and combat the deleterious effect of salt stress, the highest amount of stevioside and rebaudioside A was achieved at 0.4 g/l level of chitosan in 50 and 150 mM level of NaCl. Indeed, chitosan might be able to optimize the steviol glycoside contents in stevia accompanied by its plantation in saline soil.

Antioxidant enzymes such as superoxide dismutase, catalase and peroxidases are the main compounds to protect plants from cytotoxic effects of reactive oxygen species (ROS) in stress conditions (Ozturk et al. 2012; Zhang et al. 2013). In this study, the measured CAT activity was increased in 50 and 150 mM level of NaCl and the POX activity was incremented in all levels of NaCl treatments showing activation of defense system as ROS scavengers. By application of chitosan treatments, the highest amount of CAT activity was indicated at 0.2 g/l of chitosan in 50 mM level of NaCl as well as at 0.6 g/l of chitosan in 150 mM level of NaCl. Moreover, the highest amount of POX activity was observed in 0.2 and 0.4 g/l of chitosan in 150 mM level of NaCl as well as 0.6 g/l of chitosan in 100 mM level of NaCl. Although, no evidences have been reported related to chitosan effect on antioxidant enzymes activity in stevia, this elicitor has known to be a biological elicitor for disruption of ROS molecules. Our obtained results were similar to the results of previous researches on *Jatropha cinerea* (Hishida et al. 2014), Safflower (Jabeen and Ahmad 2013), tomato (Liu et al. 2007) and eggplant (Mandal 2010) as increasing of POX and CAT activities under salinity stress.

The increment of MDA content plays an important role in peroxidation and dilapidation of membrane lipids accompanied by membrane integrity loss (De Azevedo Neto et al. 2006). In this research, the enhancement of lipid peroxidation and MDA content were not obtained by increasing NaCl concentration. It seems that production of lower level of lipid peroxidation and less loss of electrolytes in stevia refers to the inherent salt tolerance feature of this plant. Similar result was reported by Shahverdi et al. (2019) who found stevia as moderate NaCl tolerant plant. In other view, it can be concluded that salinity tolerance of stevia is associated with higher CAT and POX activities and lower level of lipid peroxidation which exhibit better protection from oxidative damage conserving cellular membrane under different levels of salinity stresses.

Based on the obtained chitosan outcome, spraying 0.2 and 0.4 g/l of chitosan indicated the foremost concentration to decrease MDA content following by lipid peroxidation in 100 mM level of NaCl. Similar result was performed on chickpea indicating the increase of malondialdehyde (0.834 mM/g) by increasing salinity as well as decreasing MDA by application of chitosan (Mahdavi and Safari 2015). Indeed, chitosan is able to destroy ROS molecules including OH and O_2_^−^ and protect DNA molecules from peroxidation and dilapidation of membrane lipids as well as destruction of membrane integrity are achieved by increasing MDA content (Yasar et al. 2008).

## Conclusion

The results of this research leads to the conclusion that the chlorophyll pigments (such as Chl *a*, Chl *b* and total Chl) and carotenoid content decreased by all NaCl treatments. In addition, NaCl levels (50 and 150 mM) caused the increase of stevioside, rebaudioside A, protein content, CAT and POX enzyme activity except for MDA content which all of mentioned traits are the factors causing salinity stress tolerance in the plant. The increasing steviol glycosides and antioxidant enzyme activities as well as reduction of MDA levels emphasized stevia as a medium salt tolerant plant. Moreover, usage of chitosan elicitor can be applied as trustworthy method to increase the amount of salt-adaptive factors such as steviol glycosides, antioxidant enzymes and MDA. Furthermore, our data can suggest application of greater treatments of chitosan as (0.4 g/l) to optimize photosynthetic pigments, diterpene glycosides and protein content and its lower treatments (0.2 g/l) to make profitable use of CAT and POX activity and MDA content in lower level of salinity (50 mM). The resultant data can help researchers and breeders for optimal utilization of saline lands for widespread cultivation of stevia in the future.
